# Extraoral Versus Intraoral Approach for Removal of Styloid Process in Treatment of Eagle's Syndrome: A Report of Two Cases

**DOI:** 10.7759/cureus.38720

**Published:** 2023-05-08

**Authors:** Vedha Aravindan, Madhulaxmi Marimuthu, Vinod K Krishna, Alladi Sneha, Vivek Menon

**Affiliations:** 1 Oral and Maxillofacial Surgery, Saveetha Institute of Medical and Technical Sciences, Chennai, IND; 2 Oral and Maxillofacial Surgery, Saveetha Dental College and Hospital, Saveetha University, Chennai, IND; 3 Oral Oncology and Reconstructive Surgery, Saveetha Dental College and Hospital, Saveetha University, Chennai, IND; 4 Oral and Maxillofacial Surgery, Saveetha Dental College and Hospital, Saveetha University, chennai, IND

**Keywords:** styloid removal, oral and maxillofacial surgery, transpharyngeal approach, intra oral approach, extra oral approach, styloid, styloid apparatus, styloidectomy, eagles syndrome, styloid process

## Abstract

Eagle’s syndrome, a condition associated with the elongation of the styloid process or calcification of the stylohyoid ligament, is clinically characterized by throat and neck pain radiating into the mastoid region. The diagnosis can be made through a thorough history, correct clinical and pathological correlation and radiographic examination. The elongated styloid process can be treated conservatively or surgically. Conservative treatment options include transpharyngeal injections of steroids and lignocaine, nonsteroidal anti-inflammatory drugs, diazepam, and the application of heat. The surgical management of Eagle's syndrome consists of two major approaches: the transoral and the transcervical approaches. In this paper, we present a comparative study of two cases of classic bilateral elongated styloid process syndrome, treated with transcervical styloidectomy and transoral styloidectomy, their surgical time, intraoperative difficulties and complications, and recovery time.

In conclusion, the management of Eagle's syndrome requires a comprehensive approach that includes a thorough preoperative evaluation of the length of the styloid process via imaging and digital palpation. The choice of surgical approach, whether extraoral or transpharyngeal, should be based on factors such as the surgeon's experience and the patient's comorbidities, as well as the length and palpability of the styloid process. Our comparative study of two cases treated with transcervical and transoral styloidectomy demonstrated that the extraoral method offers a direct and well-controlled approach for excessive styloid processes, while the transpharyngeal approach is preferred for cases where the process can be easily identified by palpation. Therefore, proper patient selection and preoperative planning are essential to achieving successful outcomes with minimal complications.

## Introduction

Eagle's syndrome, initially described by Eagles, an otolaryngologist, in 1937, is characterized by neck and throat pain that radiates to the mastoid region. This condition arises due to elongation of the styloid process greater than 25 mm or calcification of the stylohyoid ligament [1,2]. Clinical manifestations of Eagle's syndrome include headache, dysphagia, odynophagia, pain in the mastoid region, ear pain, and dysphonia, which are often precipitated by hyperextension of the neck or rapid neck movements. Clinical examination typically reveals the obstruction of the tonsillar fossa, while the classical examination for the elongated styloid process involves probing the styloid process in the tonsillar space [3]. Eagle's syndrome can be mistaken for other facial neuralgias or temporomandibular joint disorders, but a thorough history and physical examination, including transpharyngeal palpation of the styloid process with the patient's mouth open, can help to differentiate it from these conditions. In addition, radiographic techniques such as panoramic radiographs, lateral cephalograms, posteroanterior skull views, lateral oblique views, Towne's views, and open-mouth odontoid views may be used to support the diagnosis [4].

Treatment options for Eagle's syndrome include both conservative and surgical approaches. Conservative management may involve non-steroidal anti-inflammatory drugs, heat fermentation, and trans-pharyngeal injections of lignocaine. Surgical management may involve styloidectomy through transoral or trans-cervical approaches [5]. In this paper, we present a comparative study of two cases of classic bilateral Eagle's syndrome that were treated surgically using two different approaches.

## Case presentation

Case 1

**Figure 1:**
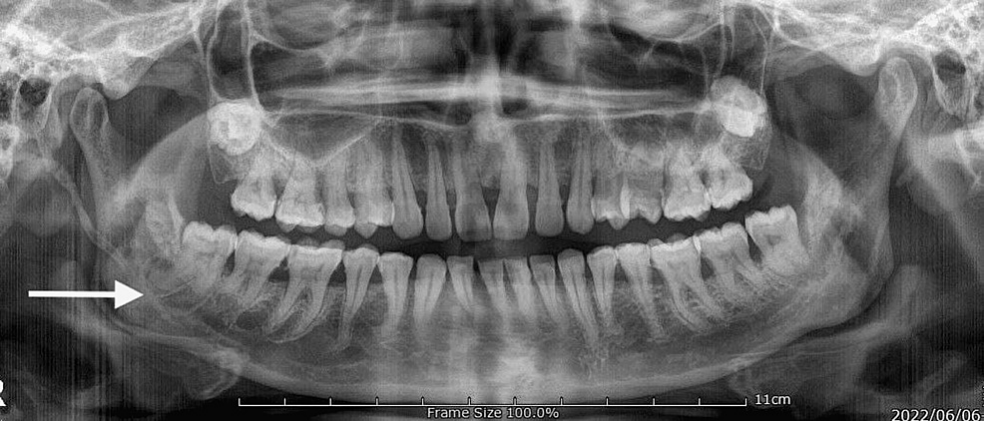
Pre-operative radiograph Represents the orthopantomogram, showing elongation of the styloid process. The arrow mark shows the elongation of the styloid process on the right side. Note that there is an elongation of the styloid process on the other side.

**Figure 2:**
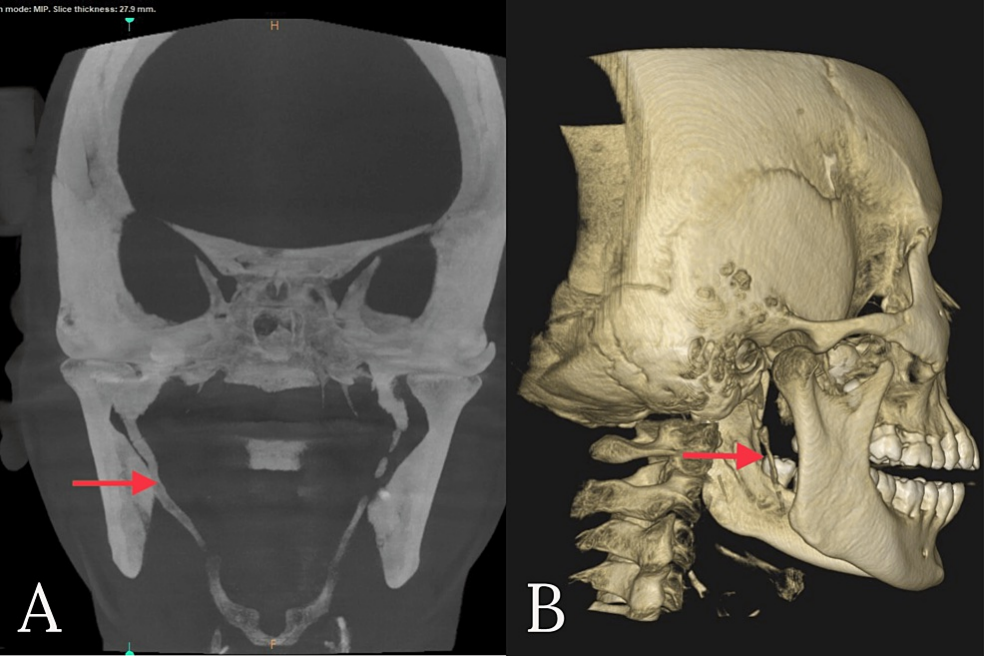
Pre-operative CBCT The arrow mark represents the elongation of the styloid process showing S curvature, the downward and forward directions in a coronal view of CBCT (A), and the 3-dimensional view of CBCT (B)

A 35-year-old female patient reported to the Department of Oral and Maxillofacial Surgery with a chief complaint of difficulty swallowing and dull pain over the throat region. The pain was insidious in onset, intermittent, and radiated to the right mastoid area and right pharyngeal region. It was relieved by bending the neck to the ipsilateral side. Associated headaches, dysphagia, discomfort on the extension of the tongue, and neck pain aggravated during swallowing were seen. The patient's past medical history was non-contributory, and the examination of the temporomandibular joint was normal. Extraoral physical examination revealed a tender, small, bony, hard projection in the right submandibular area at the anterior border of the sternocleidomastoid muscle. All third molars were impacted, and no infection or decay was seen. The bone of the styloid process could be felt when palpating the tonsillar fossa. Radiographic examination Orthopantomogram (Figure 1) demonstrated an elongated styloid process bilaterally, and this can be further confirmed by cone beam computed tomography (Figure 2), shows an elongated styloid process showing S curvature growing downwards and forward.

**Figure 3:**
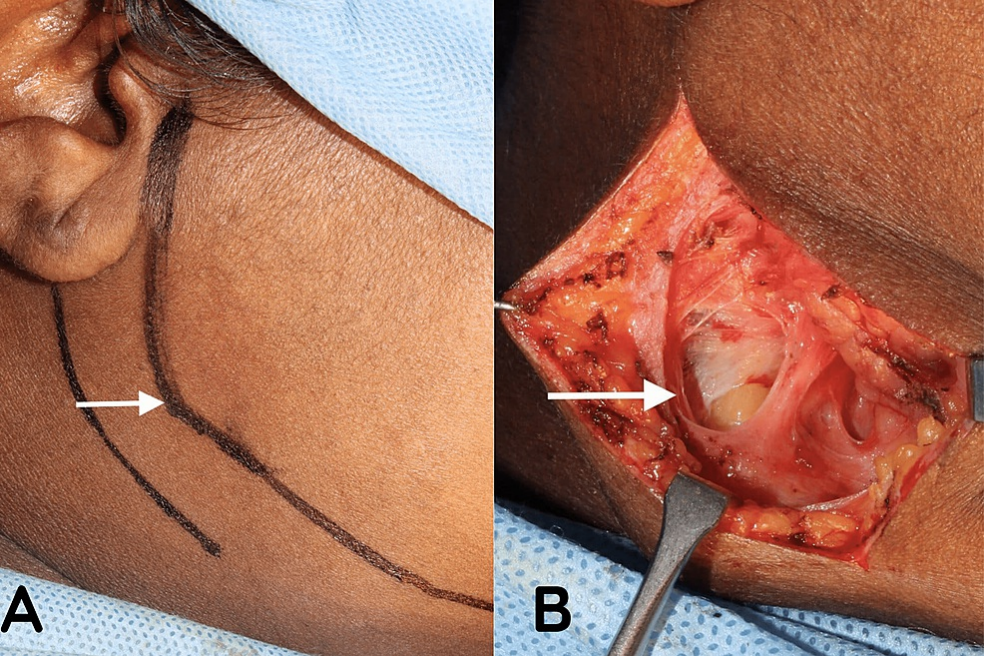
Intra-operative image The arrow mark represents the markings for an extraoral incision through the retromandibular approach for removing the styloid process (A), and the arrow on the right represents the muscle dissection and plane of orientation for styloidectomy (B).

**Figure 4:**
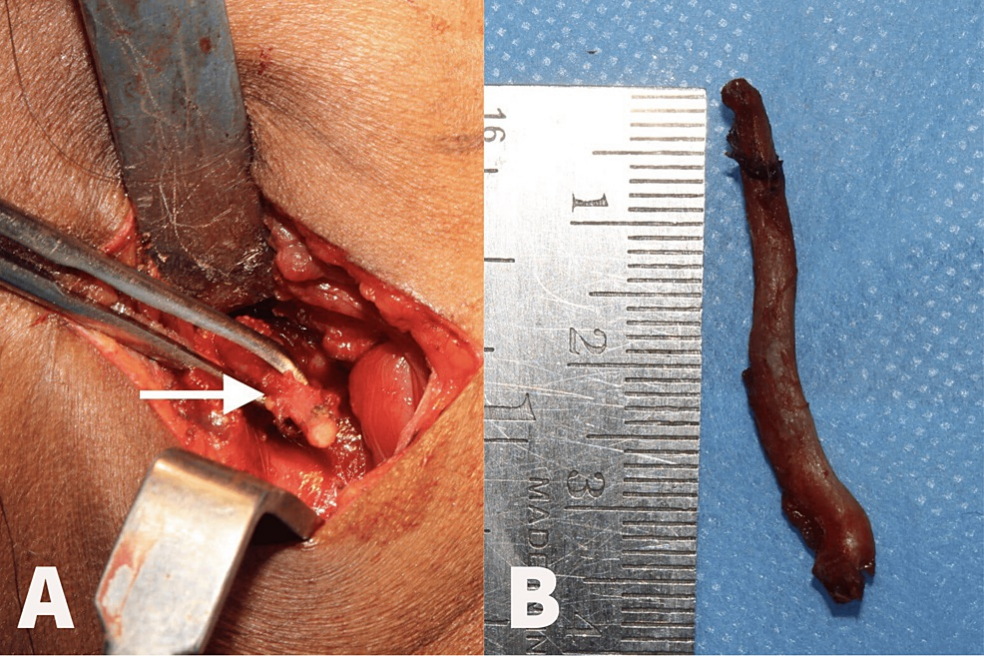
Removal of the styloid process (A) and specimen (B) The arrow mark represents the removal of the styloid process through an extraoral approach (A) and the removed styloid process of length 4 cm (B)

**Figure 5:**
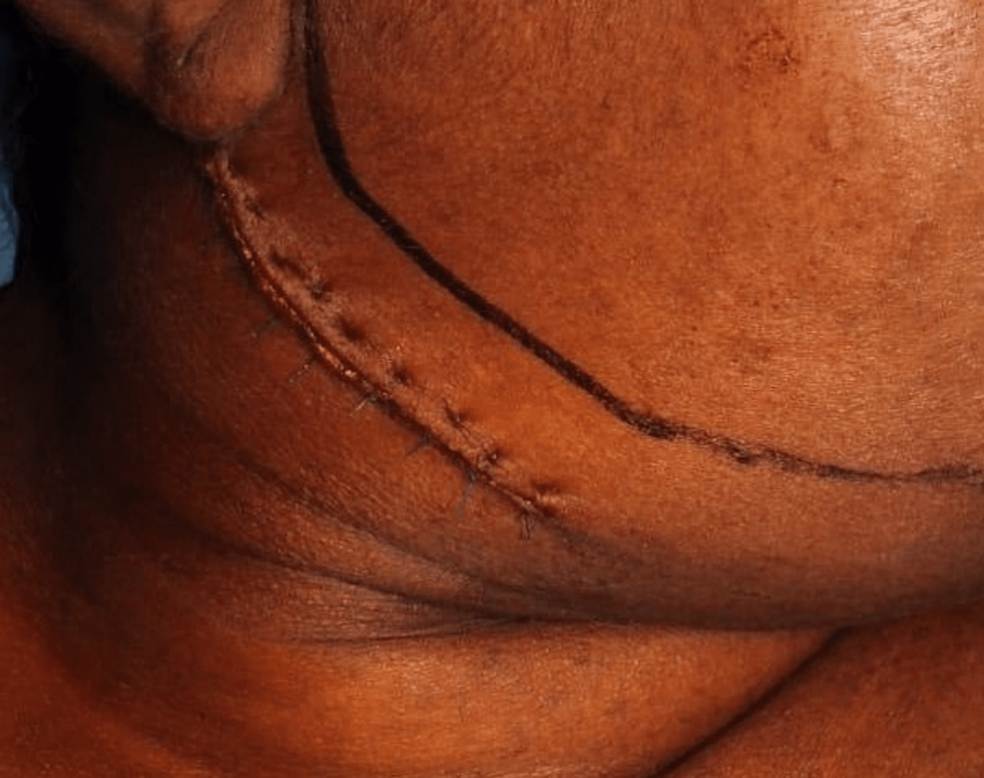
Closure Represents the closure done in layers with 3.0 polyglactin to reapproximate muscle and with 4.0 polypropylene for skin

Operative procedure: Addressing the chief complaint on the right side, surgery was planned for styloidectomy on the right side only through an extraoral approach, and after thorough investigations, the patient was scheduled for surgery under general anesthesia. An extraoral incision for the retromandibular approach was given along the ascending border of the ramus, through the skin/epidermis, and then the dermis. Then blunt dissection is done in the subplatysmal plane with a curved hemostat, dissecting the posterior portion of the masseter muscle (Figure 3). The styloid apparatus is exposed, and an incision is made along the periosteum (without disturbing the attachments) to deglove the styloid process. The naked styloid was held between hemostatic forceps and fractured, freed, and removed (Figure 4). The muscles and the mucosa were then closed with 3.0 polyglactin, and skin closure was done with 4.0 polypropylene (Figure 5). Postoperative care and follow-up were regular. Patients were given postoperative analgesics (Aceclofenac 325 mg + paracetamol 100 mg BD for three days) and antibiotics (Amoxicillin 500 mg ×3 days) and were discharged after 24 hours with appropriate postoperative instructions. Extraoral sutures were removed on the seventh postoperative day, and regular follow-ups were done. 

Case 2

**Figure 6:**
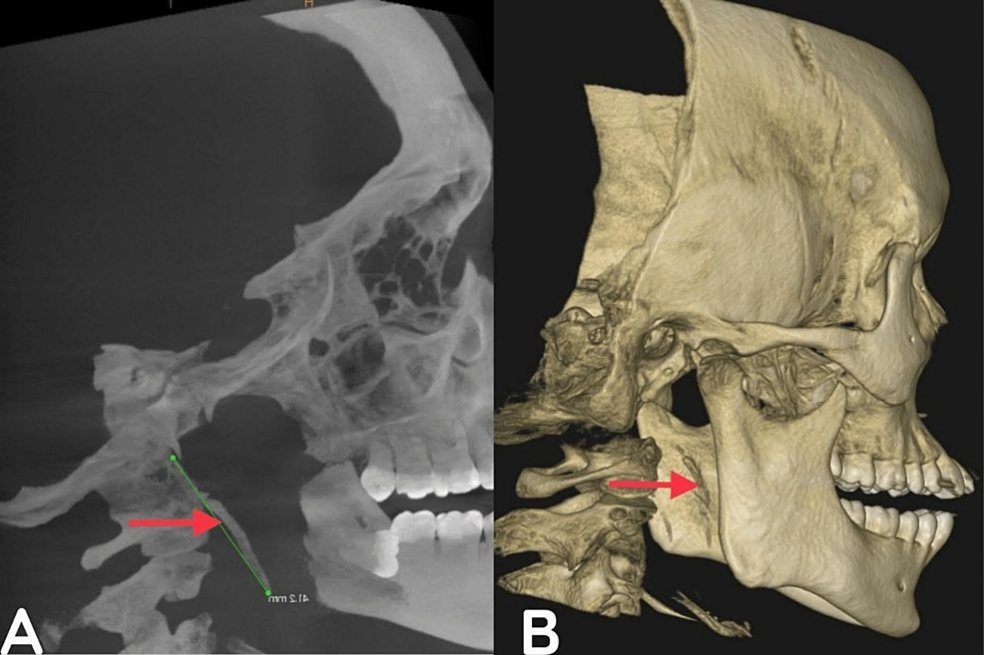
Pre-operative CBCT The arrow mark shows the elongation of the styloid process in the transverse view (A) and 3-dimensional view of CBCT (B)

A 28-year-old male patient presented to the Department of Oral and Maxillofacial Surgery with a chief complaint of intermittent pain in the right posterior tooth region, which worsened while swallowing and was associated with odynophagia. Upon intraoral examination, no impacted tooth, dental caries, or pericoronitis was detected, and the temporomandibular joint appeared normal. However, palpation of the tonsillar fossa revealed a too-long styloid process protruding from the tonsil fossa, and tenderness upon palpation indicated elongated styloid processes bilaterally. As a result, the diagnosis of a bilaterally elongated styloid process was made and confirmed with cone beam computed tomography (Figure 6), which showed an "S"-shaped bend in the bilateral elongation of the styloid process with a forward course towards the pharynx, leading to an impingement in the pharynx that was palpable intraorally. 

**Figure 7:**
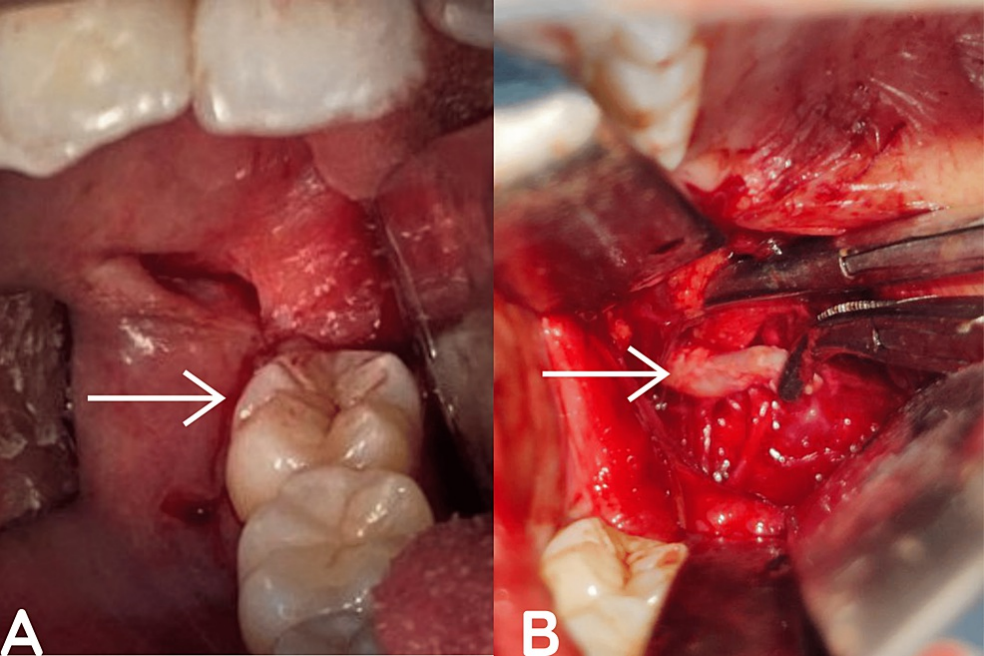
Intra-oral incision (A) and removal of the styloid process (B) The arrow mark represents the intra-oral incision for removal of the styloid process (A) and removal of the styloid process through the intra-oral approach (B)

**Figure 8:**
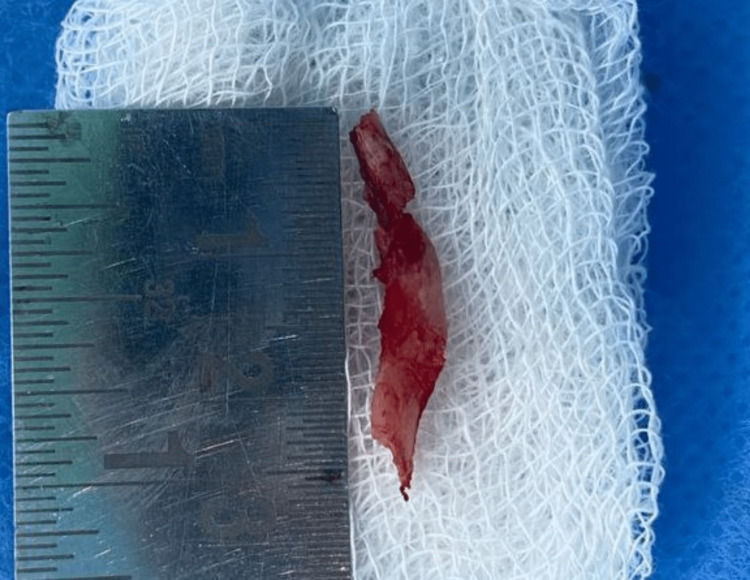
Specimen Represents the removed styloid process of length 3 cm

Operative procedure: The patient underwent a styloidectomy via the intraoral approach under general anesthesia in the operating room. The procedure involved making a transoral incision along the ascending border of the ramus (Figure 7) and performing blunt dissection using a curved hemostat. The dissection was directed posteriorly, medial to the medial pterygoid muscle, and lateral to the superior constrictor of the pharynx muscle. The styloid apparatus was then exposed, grasped between two hemostats (Figure 8), fractured, and easily removed (Figure 8). The closure was performed with 3.0 polyglactin, and postoperative analgesics and antibiotics were administered for three days. The patient was discharged on the third postoperative day and was asymptomatic at the one-year follow-up, with no complaints on either side.

## Discussion

The styloid process is a bony projection that extends 2.5 cm from the inferior part of the petrous temporal bone and is located between the internal and external carotid arteries. Several muscles and ligaments, including the stylohyoid, stylopharyngeus, styloglossus, stylohyoid ligament, and stylomandibular ligament, are attached to the styloid process. Eagle's syndrome, also known as "Stylohyoid syndrome," is characterized by an elongation of the styloid process (>25 mm), an abnormal angle, or ossification of the styloid process [6,7].

Surgical techniques used to shorten the styloid process include intraoral and extraoral approaches. In this case report, both techniques were employed, each with its advantages and drawbacks. The intraoral approach had inadequate access but had a shorter surgery duration of approximately 90 minutes. The short recovery period and self-healing of the intraoral incision allowed for quick recovery, and there was no incision or scar. The extraoral approach had excellent access but resulted in a visible scar. The surgery duration was longer at around two hours, and there was a limitation in mouth opening for two weeks postoperatively, with a gradual increase in mouth opening in the third week. The mouth opening became normal after one month [7,8]. Studies have compared the postoperative efficacy of both approaches and found no significant difference between them. The intraoral approach was deemed safer, providing less surgical trauma, less surgical time, and no scar compared to the trans-cervical approach. However, the intraoral method was found to be ineffective at controlling bleeding when vessels had been damaged due to poor visualization. Furthermore, the intraoral approach has poor visualization of the surgical field, the possibility of deep cervical infection, and the risk of neurovascular injury while attempting to leave the shortest residue of the styloid process. The extra-oral approach is criticized as having prolonged duration, morbidity, and the probability that adjacent anatomical structures could be involved [7-9].

In contrast, the extraoral technique provides greater exposure to the surgical site without obstruction from the mouth opening or the depth of the field. This technique increases visibility in cases of hemorrhage and reduces the danger of deep cervical infection. However, it has a longer operating time and can result in a neck scar, injury to the marginal mandibular branch of the facial nerve, internal carotid thrombosis, and subcutaneous cervical emphysema [10,11]. The differential diagnosis for Eagle's syndrome includes facial pain, glossopharyngeal and trigeminal neuralgia, myofascial pain dysfunction syndrome, temporal arteritis, migraine or cluster headache, pain related to impacted third molars or pericoronitis, and temporomandibular joint disorder (TMD) [12-14]. When the styloid process cannot be palpated in the tonsillar fossa, an extraoral technique can be used. Open surgery should be chosen when the styloid process follows an unconventional path, is particularly long, or extends towards the lateral cervical region to avoid extensive operation in the parapharyngeal space, a long operation time, and higher operating costs. Intraoral or extraoral approaches can also be a result of surgeon preference and experience and the patient's co-morbidity. In this study, the muscles and mucosa were closed with 3.0 polyglactin, and skin closure was done with 4.0 polypropylene in the extraoral approach, while the site was closed with 3.0 polyglactin in the intraoral approach [15,16]. Recent advances in suture materials include coatings with silver nanoparticles [17], cyanoacrylate [18], and antibiotic-coated suture materials [19], which provide better wound healing. However, considering the site of the defect, both cases were closed primarily with polyglactin, an absorbable suture material that provides better wound healing [20].

## Conclusions

To conclude, a thorough preoperative evaluation is crucial to determining the appropriate approach for the removal of the styloid process. Factors such as the length of the process, the surgeon's preference, the patient's comorbidities, and mouth opening should be taken into consideration. If the styloid process is excessive and requires surgical removal, the extraoral approach offers a direct and anatomically significant method with good visualization and controlled management of major vessel hemorrhage. However, if the process can be easily identified trans-pharyngeal by palpation, then the intraoral approach is a preferred procedure. Overall, the choice of approach should be tailored to each patient based on their specific circumstances and the surgeon's expertise.
